# HER1-Targeted ^86^Y-Panitumumab Possesses Superior Targeting Characteristics than ^86^Y-Cetuximab for PET Imaging of Human Malignant Mesothelioma Tumors Xenografts

**DOI:** 10.1371/journal.pone.0018198

**Published:** 2011-03-25

**Authors:** Tapan K. Nayak, Kayhan Garmestani, Diane E. Milenic, Kwamena E. Baidoo, Martin W. Brechbiel

**Affiliations:** Radioimmune & Inorganic Chemistry Section, Radiation Oncology Branch, National Cancer Institute, National Institute of Health, Bethesda, Maryland, United States of America; Virginia Commonwealth University, United States of America

## Abstract

Malignant mesothelioma (MM), a rare form of cancer is often associated with previous exposure to fibrous minerals, such as asbestos. Asbestos exposure increases HER1-activity and expression in pre-clinical models. Additionally, HER1 over-expression is observed in the majority of MM cases. In this study, the utility of HER1-targeted chimeric IgG_1_, cetuximab, and a human IgG_2_, panitumumab, radiolabeled with ^86^Y, were evaluated for PET imaging to detect MM non-invasively *in vivo*, and to select an antibody candidate for radioimmunotherapy (RIT).

**Methods:**

Radioimmunoconjugates (RICs) of cetuximab and panitumumab were prepared by conjugation with CHX-A’’-DTPA followed by radiolabeling with ^86^Y. The HER1 expression of NCI-H226, NCI-H2052, NCI-H2452 and MSTO-211H human mesothelioma cells was characterized by flow cytometry. *In vivo* biodistribution, pharmacokinetic analysis, and PET imaging were performed in tumor bearing athymic mice.

**Results:**

*In vivo* studies demonstrated high HER1 tumor uptake of both RICs. Significant reduction in tumor uptake was observed in mice co-injected with excess mAb (0.1 mg), demonstrating that uptake in the tumor was receptor specific. Significant differences were observed in the *in vivo* characteristics of the RICs. The blood clearance T_½_α of ^86^Y-cetuximab (0.9–1.1 h) was faster than ^86^Y-panitumumab (2.6–3.1 h). Also, the tumor area under the curve (AUC) to liver AUC ratios of ^86^Y-panitumumab were 1.5 to 2.5 times greater than ^86^Y-cetuximab as observed by the differences in PET tumor to background ratios, which could be critical when imaging orthotopic tumors and concerns regarding radiation doses to normal organs such as the liver.

**Conclusion:**

This study demonstrates the more favorable HER1-targeting characteristics of ^86^Y-panitumumab than ^86^Y-cetuximab for non-invasive assessment of the HER1 status of MM by PET imaging. Due to lower liver uptake, panitumumab based immunoconjugates may fare better in therapy than corresponding cetuximab based immunoconjugates.

## Introduction

Asbestos-related deaths have increased 400 percent in the past 20 years and the number of cases continues to increase despite awareness of asbestos-related hazards [1], [2]. Asbestos is a human mutagen and carcinogen, responsible for many pulmonary diseases including asbestosis, bronchogenic carcinoma, and malignant mesothelioma [2]. Malignant mesothelioma (MM) is a rare form of an aggressive and often treatment-resistant cancer [3]. Occupational exposure to asbestos is implicated in 70–80% of all MM. After initial diagnosis, MM has a median survival of 10–18 months [3], [4]. Conventional therapies, such as surgery, radiotherapy, and chemotherapy, do not necessarily improve overall survival. On the other hand, tremendous advances have been made regarding understanding the molecular biology of MM.

Understanding the molecular biological features of asbestos-induced MM is of critical importance. MM cells arise from the pleura or the peritoneal cavity and produce numerous growth factors, including epidermal growth factor (EGF), platelet-derived growth factor (PDGF), and transforming growth factor β (TGF-β) [3], [5], [6]. EGF is a potent mitogen for human mesothelial cells. In normal and pre-malignant animal cells of similar type, exposure to asbestos leads to autophosphorylation, increased expression of the cell surface EGF receptor (HER1) that then appears to initiate cell signaling cascades important in asbestos-induced mitogenesis and carcinogenesis [7], [8], [9].

Recent clinical studies have also shown over-expression of HER1 in MM [10], [11], [12], [13]. In an immunohistochemical (IHC) and molecular study with clinico-pathological correlations, a statistically significant correlation was observed between the expression of HER1 by IHC and corresponding mRNA levels. Secondly, HER1 mRNA levels were higher in tumor specimens than non-neoplastic pleura samples [14]. In another study comprising 71 patients, high HER1 expression was detected in 74.6% of the cases; 52.1% cases were positive for HER1 gene amplification and 45% of the cases had elevated serum HER1 [10]. In that same study, elevated serum and tissue HER1 was significantly associated with advanced disease stage, suggesting an important role of EGFR over-expression in mesothelioma [10],[11],[12],[13].

Based on the findings that HER1 is over-expressed in MM, HER1-tyrosine kinase inhibitors (TKIs) such as gefinitib and erlotinib were investigated for therapy of MM patients [15], [16]. In the study utilizing gefinitib, 97% of the patients with MM were found to have presented with disease that over-expressed HER1, the gefitinib therapy, however, was ineffective and HER1 expression did not correlate with failure-free survival [16]. Similarly, single agent erlotinib therapy was ineffective in MM, despite high expression of HER1. The authors speculated that the activation of the ERK and phosphatidylinositol 3-kinase/Akt downstream pathways as possible resistance mechanisms to erlotinib [15].

Since the majority of MMs over-express HER1, this target might prove suitable for molecular imaging and, ultimately, targeted radionuclide therapy of MM. Targeted radionuclide therapy and radioimmunotherapy (RIT) are at the forefront of molecular cancer treatment modalities that involve the use of cancer cell targeting radiopharmaceuticals, such as radiolabeled antibodies, which selectively target certain tumor cells [17], [18]. ^90^Y is one of the very promising radionuclides used for radioimmunotherapy of hematologic malignancies and solid tumors [19], [20], [21] Such radionuclide therapy outcomes will be independent of mutations in HER1 or *KRAS* domains and therefore overcome the existing limitations of conventional HER1-targeted therapy. However, since ^90^Y is a pure ß^−^-emitter, its biodistribution cannot be readily imaged for patient-specific dosimetry which is essential for pre-therapeutic treatment planning and accurate absorbed dose estimation in individual patients to mitigate radiation risks. ^111^In and ^89^Zr were used as surrogate PET radionuclides for ^90^Y, however disparities were observed in the biodistribution of these and ^90^Y labeled antibodies [22], [23]. In recent years, ^86^Y has gained popularity as an attractive surrogate for studying ^90^Y due to its half-life (14. 7 h) and positron emission which allows quantitative imaging over 2–3 days [24]. Since the chemical form is identical to ^90^Y, ^86^Y labeled antibodies have identical biodistribution to ^90^Y labeled antibodies, and therefore should enable more accurate absorbed dose estimates for ^90^Y [25]. Based on the previous experiences with ^64^Cu (half-life  = 12. 7 h) labeled antibodies in patients it is anticipated that between 0.18–0.37 GBq of the injected ^86^Y labeled antibody will result in useful quantitative images up to 2–3 d after injection [26], [27]. Therefore, in this study we sought to explore the utility of HER1-targeting ^86^Y-labeled cetuximab and panitumumab for PET imaging of MM, to assess HER1 status, and as a means to select and screen subjects for HER1-targeted radioimmunotherapy (RIT) with radionuclides such as ^90^Y for larger tumors or α-emitting radionuclides such as ^212^Pb for micrometastatic disease [28], [29], [30], [31].

In the present study, the *in vitro* characterization of four established MM cell lines for HER1 expression is described. Also detailed are the *in vivo* targeting characteristics of ^86^Y-labeled panitumumab and cetuximab in three human MM tumor xenograft models in mice for potential use in risk stratification and quantitative non-invasive imaging of HER1, and assessment of mAb uptake in MM. In addition to the development of a potential PET imaging agent, another objective of the studies described herein was the selection of a preferred antibody candidate for future RIT studies.

## Results

### *In vitro* evaluations

#### Flow cytometric analysis

Flow cytometric analysis revealed varied levels of HER1-expression for the mesothelioma cell lines evaluated (Table S1). NCI-H226 had the highest mean fluorescence intensity (MFI), whereas NCI-H2452 had the lowest MFI (Table S1). Panitumumab and cetuximab demonstrated comparable *in vitro* binding characteristics for each cell type, as evidenced by the percentage of cells stained with each of the mAb.

#### Radiochemistry

The ^86^Y labeled RICs were successfully prepared with radiochemical yields ranging from 60-75%, specific activity exceeding 2 GBq/mg, and with acceptable *in vitro* receptor-specificity as previously described [29], [30].

### *In vivo* evaluations

#### Biodistribution studies

**Figure 1:**
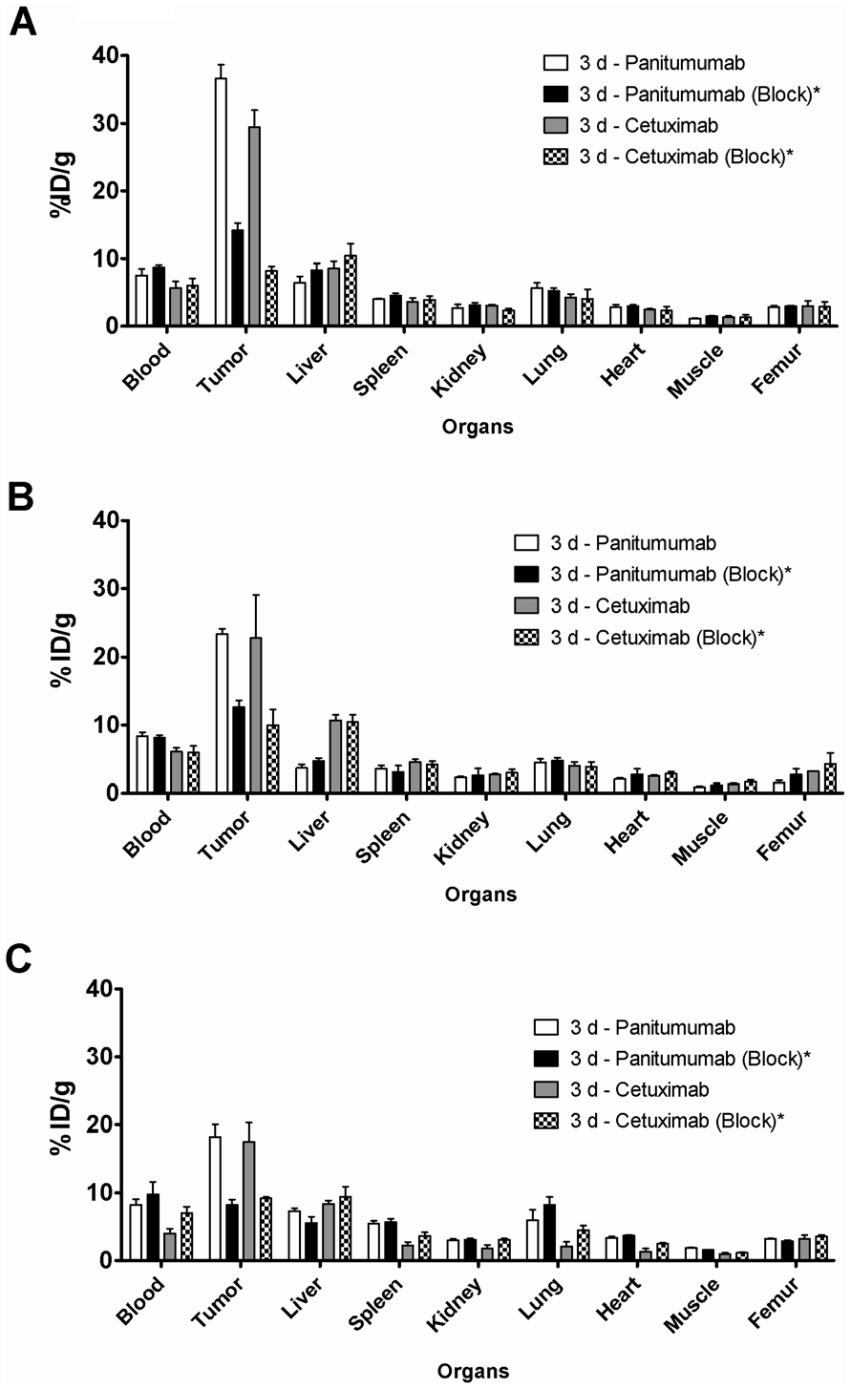
HER1-specificity of ^86^Y-CHX-A’’-DTPA-panitumumab and ^86^Y-CHX-A’’-DTPA-cetuximab. Receptor-meditated uptake of ^86^Y-CHX-A’’-DTPA-panitumumab and ^86^Y-CHX-A’’-DTPA-cetuximab in selected organs of female athymic (NCr) *nu/nu* mice bearing NCI-H226 (**A**), MSTO-211H (**B**) and NCI-H2052 tumor xenografts (**C**). Biodistribution data were obtained 3 d after injection. All values are expressed as % ID/g. Data represent the mean value ± SEM from at least three determinations. ^*^Receptor blocking studies were performed by co-injecting 0.1 mg mAb with the radiotracer. Values obtained from the blocking studies were significantly lower than the unblocked studies (*p*<0.05) demonstrating receptor-mediated accumulation in the tumors.

**Figure 2:**
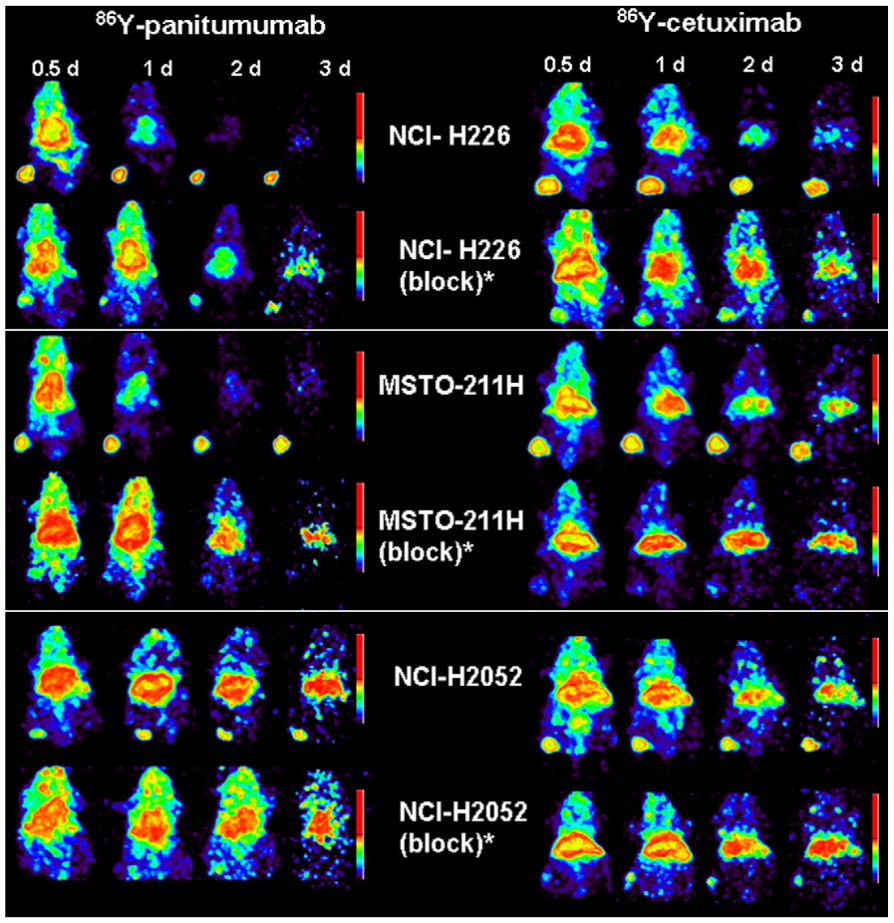
PET imaging of mesothelioma with ^86^Y-CHX-A’’-DTPA-panitumumab and ^86^Y-CHX-A’’-DTPA-cetuximab. Representative reconstructed and processed maximum intensity projections of female athymic (NCr) *nu/nu* mouse bearing NCI-226, MSTO-211H and NCI-H2052 tumor xenografts. Mice represented in the images were injected i.v. via the tail vein with 1.7–1.9 MBq/<5 µg of the radioimmunoconjugate or co-injected with 0.1 mg excess mAb. The scale represents % maximum and minimum threshold intensity. ^*^Receptor blocking studies were performed by co-injecting 0.1 mg excess mAb with the corresponding radioimmunoconjugate.

In mice bearing the NCI-H226 tumor xenograft, significant decreases in the blood pool activity was observed over a 4 d time period for both RICs (Table 1). For ^86^Y-CHX-A’’-DTPA-panitumumab, the blood % ID/g decreased from 12.06±1.28 at 1 d to 6.94±1.09% ID/g at 4 d, a 43% decrease. ^86^Y-CHX-A’’-DTPA-cetuximab showed an even greater decrease beginning with a blood %ID/g of 11.70±1.44% ID/g at 1 d and ending with 3.40±0.60% ID/g at 4 d injection, 29% of the initial level. Meanwhile, the tumor uptake increased over a 4 d time period for both RICs (Table 1). The tumor %ID/g of ^86^Y-CHX-A’’-DTPA-panitumumab, was 23.13±3.36 at 1 d which increased to 33.18±1.84 at 4 d; similarly for ^86^Y-CHX-A’’-DTPA-cetuximab, the tumor %ID/g was 21.24±1.90% ID/g at 1 d and increased to 28.93±3.35 4 d post-injection. The ^86^Y-CHX-A’’-DTPA-panitumumab and ^86^Y-CHX-A’’-DTPA-cetuximab uptake in all the three tumor models was HER1-mediated as demonstrated by receptor-blocking experiments performed by co-injecting 0.1 mg of the corresponding unlabeled mAb (Fig. 1). In mice bearing NCI-H226 (Fig. 1A), MSTO-211H (Fig. 1B) or NCI-2052 (Fig. 1C) tumors, the tumor % ID/g at 3 d was 36.5±2.1, 23.4±0.9 and 18.2±1.8, respectively, after i.v. injection of ^86^Y-CHX-A’’-DTPA-panitumumab. The corresponding tumor % ID/g in mice co-injected with 0.1 mg of panitumumab was 14.1±1.1, 12.7±1.0 and 8.3±0.8, respectively, at the same time point, thus demonstrating specificity of the ^86^Y-CHX-A’’-DTPA-panitumumab. Similarly, for ^86^Y-CHX-A’’-DTPA-cetuximab, the tumor % ID/g at 3 d was 29.4±2.5, 22.8±6.2 and 19.1±1.9, respectively, and the corresponding tumor % ID/g in mice co-injected with 0.1 mg of cetuximab was 8.2±0.7, 10.0±2.3 and 9.2±0.3, respectively, at the same time point. The values were significantly different (*p* <0.05) between unblocked and blocked groups for each tumor type and for both RICs. Although both RICs demonstrated HER1-mediated targeting characteristics, subtle and noteworthy differences in organ uptake were observed at different time points after the injection (Table 1). At 1 and 2 d after injection, the liver uptake of ^86^Y-CHX-A’’-DTPA-cetuximab was significantly greater (*p* = 0.007 and 0.040 at 1 and 2 d, respectively) than the liver uptake of ^86^Y-CHX-A’’-DTPA-panitumumab in the same tumor model (Table 1). However, at 4 d after injection, the blood, spleen, kidney, lung and heart uptake of ^86^Y-CHX-A’’-DTPA-panitumumab was significantly greater than the uptake of ^86^Y-CHX-A’’-DTPA-cetuximab in those organs in the same tumor model (Table 1). Inter-tumor differences were observed too. The liver uptake of ^86^Y-CHX-A’’-DTPA-panitumumab was greater in mice bearing NCI-H226 tumors than in mice bearing MSTO-211H tumors; however, the same phenomenon was not observed with ^86^Y-CHX-A’’-DTPA-cetuximab (Fig. 2A and B).

**Table 1:** Biodistribution of ^86^Y-CHX-A’’-DTPA-panitumumab and ^86^Y-CHX-A’’-DTPA-cetuximab.

Organs	1 d		2 d		3 d		4 d	
	Panitumumab	Cetuximab	Panitumumab	Cetuximab	Panitumumab	Cetuximab	Panitumumab	Cetuximab
**Blood**	12.06±1.26	11.70±1.44	8.59±1.62	8.16±0.88	7.55±0.92	5.66±0.99	6.94±1.09	3.40±0.60#
**Tumor**	23.13±3.36	21.24±1.90	27.23±2.18	24.69±1.99	36.55±2.04	29.43±2.53	33.18±1.84	28.93±3.35
**Liver**	7.38±0.83	13.15±1.21#	6.64±0.61	9.53±0.93#	6.35±0.82	8.77±0.91	5.04±0.32	5.90±0.82
**Spleen**	4.69±1.04	3.96±0.51	4.75±0.68	3.80±0.49	4.05±0.02	3.58±0.59	4.22±0.28	1.48±0.25#
**Kidney**	3.45±0.71	3.61±0.46	2.58±0.18	2.55±0.21	2.69±0.56	3.06±0.16	2.35±0.18	1.53±0.15#
**Lungs**	5.96±1.39	5.12±0.35	5.03±2.40	3.08±0.23	5.67±0.79	4.25±0.50	4.45±0.17	1.92±0.36#
**Heart**	3.55±0.76	3.75±0.27	2.29±0.39	1.96±0.12	2.81±0.41	2.50±0.09	2.45±0.16	1.24±0.14#
**Muscle**	1.75±0.21	1.54±0.07	1.60±0.58	1.02±0.13	1.11±0.07	1.34±0.19	1.00±0.13	0.63±0.11
**Femur**	2.75±0.18	3.04±0.25	2.51±0.54	2.43±0.13	2.85±0.20	2.97±0.76	2.52±0.23	2.70±0.55
**Tail**	2.12±0.42	2.10±0.05	1.49±0.54	2.27±0.24	1.59±0.27	2.05±0.14	2.11±0.18	1.84±0.29

*In vivo* biodistribution of ^86^Y-CHX-A’’-DTPA-panitumumab and ^86^Y-CHX-A’’-DTPA-cetuximab injected i.v. via tail vein of female athymic (NCr) *nu*/*nu* mice bearing NCI-H226 tumor xenograft. Biodistribution data were obtained at 1, 2, 3 and 4 d after injection. All values are expressed as % ID/g. Data represents the mean value ± SEM from at least four determinations.

# Values obtained from ^86^Y-CHX-A’’-DTPA-panitumumab and ^86^Y-CHX-A’’-DTPA-cetuximab were significantly different from each other (*p*<0.05).

#### PET imaging studies and pharmacokinetic analysis

**Figure 3:**
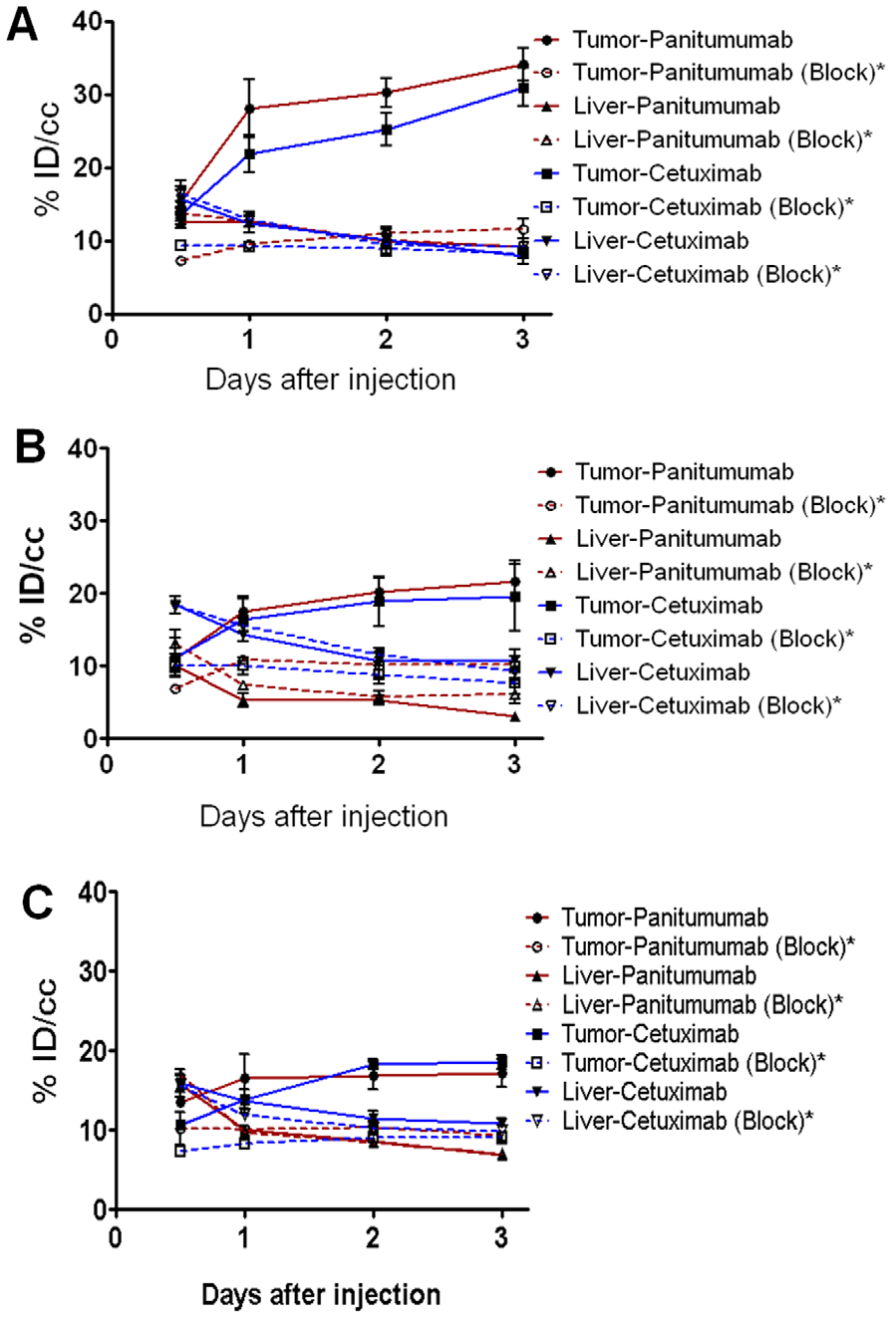
Time-activity curves obtained from quantitative PET imaging of mesothelioma with ^86^Y-CHX-A’’-DTPA-panitumumab and ^86^Y-CHX-A’’-DTPA-cetuximab. PET generated time-activity curves in mice bearing (A) NCI-H226, (B) MSTO-211H and (C) NCI-H2052 tumor xenografts. ^*^Receptor blocking studies were performed by co-injecting 0.1 mg excess mAb with the corresponding radioimmunoconjugate.

Small animal PET imaging studies were performed in female athymic mice bearing NCI-H226, MSTO-211H and NCI-H2052 tumor xenografts injected with 1.7–1.9 MBq of RIC or RIC co-injected with 0.1 mg excess of the corresponding mAb (Figure 2). Tumors were clearly visualized in maximum intensity projections of mice imaged from 0.5 to 3 d after injection of either of the RICs. The tumor-to-background ratios improved over the period primarily due to the decrease and clearance of the radioactivity in blood, liver and background while the tumor uptake increased. In contrast, when 0.1 mg of excess mAb was co-injected with its corresponding RIC, tumors were poorly visualized due to receptor-specific blockage, demonstrating the HER1-specificity of both radioimmunoconjugates also shown in quantitative information obtained from PET studies (Figure 3). Significant differences were found between the liver uptake of mice injected with ^86^Y-CHX-A’’-DTPA-panitumumab and mice injected with ^86^Y-CHX-A’’-DTPA-cetuximab, particularly in the NCI-H226 and MSTO-211H tumor models. The liver clearance of the ^86^Y-CHX-A’’-DTPA-cetuximab was slower than that of ^86^Y-CHX-A’’-DTPA-panitumumab therefore resulting in lower tumor-liver ratios than ^86^Y-CHX-A’’-DTPA-panitumumab.

Pharmacokinetic analyses performed on biodistribution and PET derived values, and from values obtained from serial blood sampling revealed further differences between ^86^Y-CHX-A’’-DTPA-panitumumab and ^86^Y-CHX-A’’-DTPA-cetuximab (Table 2). The blood T_½_α for ^86^Y-CHX-A’’-DTPA-panitumumab was significantly slower than that of ^86^Y-CHX-A’’-DTPA-cetuximab; however, no significant difference was observed between the two RICs in the T_½_β phase. The ^86^Y-CHX-A’’-DTPA-panitumumab blood AUC_[0→4]_ values were slightly greater than ^86^Y-CHX-A’’-DTPA-cetuximab values, however the difference was not statistically significant. For both RICs, the tumor AUC_[0→4]_ value were highest for mice bearing NCI-H266 tumor xenografts and lowest for mice bearing NCI-H2052 tumor xenografts (Table 2). For each tumor model, the ^86^Y-CHX-A’’-DTPA-cetuximab liver AUC_[0→4]_ values were significantly greater than that of ^86^Y-CHX-A’’-DTPA-panitumumab. The ^86^Y-CHX-A’’-DTPA-panitumumab tumor AUC_[0→4]_: liver AUC_[0→4]_ ratio values were 1.7, 2.5 and 1.4 times greater than values obtained from ^86^Y-CHX-A’’-DTPA-cetuximab in mice bearing NCI-H226, MSTO-211H and NCI-H2052, respectively (Table 2). The mean residence times were identical for all of the tumor models and RICs. For both RICs, PET derived tumor AUC_[0→3]_ values were significantly greater than corresponding tumor AUC_[0→3]_ values derived from blocking experiments with 0.1 mg excess mAb co-injection (Table 2 and Figure 3), thus again demonstrating HER1-mediated tumor accumulation over the 3 d study period. The organ uptake values quantified by PET were closely related (r^2^ = 0.92, *p* = 0.90, n = 76) to values determined by *ex vivo* biodistribution studies.

**Table 2:** Pharmacokinetic characteristics of ^86^Y-CHX-A’’-DTPA panitumumab and ^86^Y-CHX-A’’-DTPA-cetuximab.

Pharmacokinetic characteristics	NCI-H226		MSTO-211H		NCI-H2052	
^86^Y labeled antibody	Panitumumab	Cetuximab	Panitumumab	Cetixumab	Panitumumab	Cetuximab
***In vitro*** ** expression as MFI (%)**	375.4 (99.85)	345.4 (99.7)	217.2 (87.2)	224.4 (79.9)	330.5 (93.0)	337.5 (90.3)
**Blood clearance (h)**	α- t_1/2_ = 3.1±1.4β- t_1/2_ = 62.1±16.1	α- t_1/2_ = 0.9±0.2#β- t_1/2_ = 43.5±11.5	α- t_1/2_ = 3.0±0.9β- t_1/2_ = 58.1±10.2	α- t_1/2_ = 1.1±0.1#β- t_1/2_ = 47.8±11.9	α- t_1/2_ = 2.6±1.2β- t_1/2_ = 86.9±24.3	α- t_1/2_ = 0.9±0.3#β- t_1/2_ = 46.1±9.8
**Blood AUC_[0→4]_ (%ID^.^d^.^g^−1^)**	26.6±1.5	21.7±2.3	30.3±2.1	29.7±1.9	30.3±1.8	29.7±3.1
**Tumor AUC_[0→4]_ (%ID^.^d^.^g^−1^)**	105.7±5.8	90.4±8.2	69.8±8.5	63.0±4.2	60.6±3.4	58.6±2.9
**Liver AUC_[0→4]_ (%ID^.^d^.^g^−1^)**	24.2±1.2	35.1±3.2#	18.2±1.1	40.6±3.3#	29.7±2.1	40.7±3.4#
**Tumor PET AUC_[0→3]_ (%ID^.^d^.^cc^−1^)**	72.3±4.8	60.7±5.5	46.9±5.5	43.7±3.2	41.2±3.9	40.6±2.6
**Tumor PET AUC_[0→3]_ (%ID^.^d^.^cc^−1^)** *	26.1±1.2	22.3±0.4	25.0±1.2	22.5±1.0	25.0±0.9	21.6±1.1
**Tumor AUC_[0→4]_: Blood AUC_[0→4]_**	4.0	4.2	2.3	2.1	2.0	2.0
**Tumor AUC_[0→4]_: Liver AUC_[0→4]_**	4.4	2.6	3.8	1.5	2.0	1.4
**Tumor AUMC_[0→4]_ (%ID^.^ d^2.^g^-1^)**	253.6±16.2	216.8±18.4	172.5±18.1	151.0±11.2	152.3±8.1	146.4±9.9
**Tumor MRT (d)**	2.4	2.4	2.5	2.4	2.5	2.5

Pharmacokinetic characteristics of ^86^Y-CHX-A’’-DTPA-panitumumab and ^86^Y-CHX-A’’-DTPA-cetuximab injected i.v. via tail vein of female athymic (NCr) *nu*/*nu* mice bearing NCI-H226, MSTO-211H and NCI-H2052 tumor xenografts. Data represent the mean values from three to six determinations.

*Receptor blocking studies were performed by co-injecting 0.1 mg mAb with the radiotracer. Values obtained from the blocking studies were significantly lower than the unblocked studies (*p*<0.05) demonstrating receptor-mediated accumulation in the tumors.

# Values obtained from ^86^Y-CHX-A’’-DTPA-panitumumab and ^86^Y-CHX-A’’-DTPA-cetuximab were significantly different from each other (*p*<0.05).

## Discussion

Information available from the Surveillance, Epidemiology and End Results (SEER) confirms that the incidence of mesothelioma has increased in recent years from almost nil to the current 2500–3000 cases per year in the USA [1]. Diagnosis of MM is difficult and current treatments do not provide significant improvements in survival. Computed Tomography (CT) has been widely used as the primary imaging modality for the diagnosis, staging, and monitoring of therapeutic response in MM. More recently, MRI and PET imaging with FDG have gained popularity for imaging MM because of the excellent resolution and superiority in the differentiation of malignant from benign disease [32], [33], [34]. A prospective study comprising 32 patients, 19 with malignant and 13 with benign disease, found that FDG PET had a high negative predictive value of 92%. FDG PET imaging showed an absence of FDG uptake, and correctly classified 31/35 benign lesions. Nevertheless, the usefulness of FDG is limited by its uptake in inflammatory cells such as macrophages and activated lymphocytes, which can cause false-positive results as seen in cases of parapneumonic effusion, tuberculous and uraemic pleural disease [35], [36]. In spite of recent advances in the diagnosis of MM, therapeutic outcomes have not greatly improved. Surgical resection and adjuvant radiation therapy remain the mainstay of treatment for patients with resectable MM [37].

Occupational exposure to asbestos remains the major risk factor for MM. This exposure has been demonstrated to be associated with increased HER1 activation and expression [7], [8], [9]. Therefore, HER1-targeted imaging can play a complimentary role in a better understanding of asbestos-induced mesothelioma. While traditional targeting of HER1 for therapy has not been successful, it is feasible that HER1 binding molecules could be used as vectors to effectively deliver imageable or cytotoxic radioactive payloads differentially to MM cells to potentially improve diagnostic as well as therapeutic outcomes. Towards this end, the *in vivo* targeting characteristics of two HER1-binding monoclonal antibodies, cetuximab and panitumumab, each labeled with ^86^Y, were comparatively evaluated as potential diagnostics by PET imaging, and to select a potential candidate for evaluation in monoclonal antibody targeted RIT applications.

*In vitro* HER1 expression was observed in four MM cell lines evaluated (Table S1) similar to the clinical findings of HER1 over-expression in majority of MM [10], [11], [12], [13]. HER1-specific tumor targeting was observed in all three xenograft tumor models evaluated (Figs. 1, 2, and Table 2) supporting the hypothesis that HER1 targeting can be used for imaging and radionuclide therapy of MM. Although both radiolabeled cetuximab and panitumumab demonstrated *in vivo* HER1-targeting characteristics, disparities were observed with blood clearance and non-target organ uptake (Table 1 and 2). Cetuximab is a chimeric IgG_1_ mAb, whereas panitumumab is a fully human IgG_2_ mAb and binds to a different epitope of the HER1 antigen than cetuximab. Antibodies are usually cleared through their interaction with the Fc receptors expressed on cells of the reticuloendothelial system [38], [39]. The slower first-phase blood clearance of ^86^Y-CHX-A’’-DTPA-panitumumab may be attributed to the fact that panitumumab is an IgG_2_ whereas cetuximab is an IgG_1_. IgG_2_ antibodies have lower affinity and binding to the Fc-gamma receptors than the IgG_1_ and therefore are cleared more slowly by this mechanism [38], [39]. As observed in the biodistribution (Table 1) and PET imaging studies (Fig. 2 and Table 2), the liver uptake and accumulation of ^86^Y- CHX-A’’-DTPA-cetuximab was significantly greater than that of ^86^Y- CHX-A’’-DTPA-panitumumab in all three xenograft tumor models, and as a result, tumor to liver ratios were better for ^86^Y- CHX-A’’-DTPA-panitumumab than ^86^Y- CHX-A’’-DTPA-cetuximab (Table 2). For ^90^Y- RIT, the data obtained from this pre-clinical study indicate that RIT with ^90^Y- CHX-A’’-DTPA-cetuximab and ^90^Y- CHX-A’’-DTPA-panitumumab will result in similar tumor accumulation; however, that same data also suggests that ^90^Y- CHX-A’’-DTPA-cetuximab will result in higher radiation doses to the liver than ^90^Y- CHX-A’’-DTPA-panitumumab due significantly greater cumulative activity in liver (presented as AUC in table 2). Therefore, ^90^Y- CHX-A’’-DTPA-panitumumab may be a more favorable candidate for RIT than ^90^Y- CHX-A’’-DTPA-cetuximab due to higher tumor:liver that may result in lower radiation doses to the normal organs than ^90^Y- CHX-A’’-DTPA-cetuximab. Previous clinical study with ^111^In labeled 225 (murine version of cetuximab) suggests the presence of HER1 receptor in the liver based on the dose-dependent liver uptake and clearance of the ^111^In labeled murine 225. However, a study performed with radiolabeled chimeric mAb, C225 (cetuximab) concluded that the residence time in the liver appeared to be longer in patients with cold loading than in those without. One explanation could indeed be that the liver does not have C225 binding sites, but simply metabolically extracts whatever is not taken up elsewhere in the body. In the pre-clinical study performed in the report, the uptake in liver was not blocked by co-injecting excess cetuximab and panitumumab, suggesting the lack of cetuximab and panitumumab binding sites in mouse liver, which in part concurs with the information provided by the manufacturer of cetuximab, ImClone Systems. Therefore, the differences in liver zzzuptake may be a function of radiometabolities and/or Fc-gamma interactions of cetuximab and panitumumab. These differences can also have a significant impact for targeting intrapleural and intraperitoneal MM with respect to signal to noise ratios as well as radiation doses delivered to the liver, particularly in the setting of radionuclide therapy.

For this reason, panitumumab presents as a better alternative than cetuximab for HER1-targeted imaging and RIT. The HER1- targeting characteristics of radiolabeled panitumumab shown here points to its potential as a great diagnostic tool for detection and staging of MM. The results also point to the potential of panitumumab as a vehicle for delivering therapeutic radioactivity to HER1-expressing MM tumors. This approach to MM therapy should improve outcomes for HER1 over-expressing tumors that have not responded to classical HER1 therapy with TKIs and monoclonal antibodies due to resistance.

### Conclusions

In this study, the more favorable HER1-targeting characteristics of ^86^Y- CHX-A’’-DTPA-panitumumab than ^86^Y- CHX-A’’-DTPA-cetuximab for non-invasive staging and assessment of the HER1 status of MM has been demonstrated. HER1-targeted immunoPET can be complimentary to CT and MRI for diagnosis and prognosis of MM. Valuable molecular information on further understanding the role of HER1 in asbestos-induced MM may also be garnered. In conclusion, the strategy to target asbestos-induced HER1 over-expression for molecular imaging and radionuclide therapy warrants further investigation for clinical translation and improved clinical outcomes and management of MM.

## Materials and Methods

### Cell lines and tissue culture

NCI-H226, NCI-H2052, NCI-H2452 and MSTO-211H human mesothelioma cells were purchased from American Type Culture Collection (Manassas, VA). All cell lines were grown as a monolayer at 37°C, in a humidified atmosphere of 5% CO_2_ and 95% air. Cells were cultured in RPMI-1640 media containing 2 mM L-glutamine, 10 mM HEPES, 1 mM sodium pyruvate, 4.5 g/L glucose, and 1.5 g/L sodium bicarbonate. All media were additionally supplemented with 10% FetalPlex (Gemini Bio-Products, Inc, Woodland, CA, USA). Media and supplements were obtained from Invitrogen (Carlsbad, CA, USA) and Lonza (Walkersville, MD, USA).

### Flow-Cytometric Analysis

HER1 expression of the mesothelioma cell lines was evaluated by standard flow-cytometric techniques [40]. Briefly, cells were trypsinized, pelleted at 1,500× g for 10 min and re-suspended in phosphate-buffered saline (PBS; pH 7.2) containing 1% bovine serum albumin (BSA). The cells (1×10^6^ cells in 100 µL of 1% BSA in PBS) were added to 12×75 mM polypropylene tubes (Falcon Labware, Franklin Lakes, NJ) along with 1 µg of cetuximab (Erbitux: Bristol-Meyers Squibb Co, Princeton, NJ) or panitumumab (Vectibix: Amgen, Thousand Oaks, CA) in 100 µL. The cells were incubated for 1 h at 4°C, washed three times by adding 2 mL of 1% BSA in PBS, pelleting the cells at 1,000× g for 5 min and decanting the supernatant. Following the last wash, 100 µL of FITC-labeled goat anti-human IgG (50 µg/mL; Kirkegaard and Perry, Gaithersburg, MD) was added to the cells and incubated for an additional 1 h at 4°C. The cells were washed three times as before and analyzed (10,000 events) using a FACScalibur (BD Biosciences, San Jose, CA) with CellQuest software. HuM195, an anti-CD33 mAb kindly provided by Dr. Michael McDevitt at Memorial Sloan-Kettering Cancer Center, served as a control mAb.

### Preparation of radioimmunoconjugates

The ^86^Y was produced by the previously described ^86^Sr(p,n)^86^Y reaction using a SrCO_3_ target [29], [41]. The preparation and quality control of ^86^Y-CHX-A’’-DTPA-panitumumab and ^86^Y-CHX-A’’-DTPA-cetuximab conjugates was performed as previously described [29], [30].

### Animal and tumor models

All animal studies were performed in accordance with the NIH guidelines for the humane use of animals and all procedures were reviewed and approved by the National Cancer Institute Animal Care and Use Committee (Protocol ID: ROB-104/5). Groups of 5–8 week old female athymic *nu/nu* mice (Charles River Laboratory, Wilmington, DE) were injected subcutaneously with 2–4×10^6^ MSTO-211H, 6–10×10^6^ NCI-H226, or 6–10×10^6^ NCI-H2052 cells in 200 µL medium containing 20% matrigel.

### Biodistribution and pharmacokinetic studies

Tumor bearing female athymic mice were intravenously (i.v.) injected with 0.4–0.6 MBq (<5 µg) of ^86^Y-CHX-A’’-DTPA-cetuximab or ^86^Y-CHX-A’’-DTPA-panitumumab. To demonstrate HER1-specificity, excess mAb (0.1 mg) was co-injected with the corresponding radioimmunoconjugate (RIC) into an additional set of mice bearing each of the tumor xenografts. At the desired time points, the animals were sacrificed by CO_2_ inhalation. Tumor, blood and selected organs were harvested, wet-weighed, and the radioactivity measured in a Wizard 1480 gamma counter (PerkinElmer, Shelton, CT). The percent injected dose per gram (% ID/g) of tissue was calculated by comparison with standards representing 10% of the injected dose per animal. Non-compartmental pharmacokinetics was performed to determine area under the curve (AUC), area under the first moment curve (AUMC) and the mean residence time (MRT) using trapezoidal integration analysis [42]. The sample size for biodistribution study was equal to or greater than four animals per group.

### PET imaging studies

Small animal PET studies were performed using the ATLAS (Advanced Technology Laboratory Animal Scanner) at the National Institutes of Health, Bethesda, MD, USA. Whole body imaging studies (6 bed positions, total acquisition time of 1 h per mouse) were carried out on mice anesthetized with 1.5–1.7% isoflurane on a temperature-controlled bed as previously described [29]. Tumor bearing female athymic mice were injected i.v. with 1.7–1.9 MBq (<5 µg) of ^86^Y-CHX-A’’-DTPA-cetuximab or ^86^Y-CHX-A’’-DTPA-panitumumab. To determine HER1-specificity, excess unmodified mAb (0.1 mg) was co-injected with the corresponding RIC. Phantom studies, image acquisition, processing and analysis was performed as previously described [29]. After imaging, the mice were euthanized and biodistribution studies were performed to determine the correlation between PET-assessed *in vivo* % ID/cm^3^ and biodistribution determined *ex vivo* % ID/g. The sample size for PET imaging study was equal to or greater than three animals per group.

### Statistical Analysis

All numerical data were expressed as the mean of the values ± the standard error of mean (SEM). Graphpad Prism version 5 (San Diego, CA, USA) was used for statistical analysis. A *p* value less than 0.05 was considered statistically significant.

## Supporting Information

Table S1 Relative *in vitro* expression of HER1 in human mesothelioma cells determined by FACS based assay. MFI  =  mean fluorescence intensity.
(DOC)
