# Supplementary material for: HER1-Targeted 86Y-Panitumumab Possesses Superior Targeting Characteristics than 86Y-Cetuximab for PET Imaging of Human Malignant Mesothelioma Tumors Xenografts
Source: PLoS One. 2011 Mar 25;6(3):e18198. doi: 10.1371/journal.pone.0018198 (PMC3064677; doi:10.1371/journal.pone.0018198)
Supplement: Table S1 — Relative in vitro expression of HER1 in human mesothelioma cells determined by FACS based assay. MFI = mean fluorescence intensity. (DOC) [file pone.0018198.s001.doc]

**Supplemental information**

Nayak et al. HER1-targeted 86Y-panitumumab possesses superior targeting characteristics than 86Y-cetuximab for PET imaging of human malignant mesothelioma tumors xenografts.

|  | Cetuximab | | Panitumumab | | HuM195 | |
| --- | --- | --- | --- | --- | --- | --- |
| Cell Line | MFI | % | MFI | % | MFI | % |
|  |  |  |  |  |  |  |
| MSTO-211H | 224.4 | 79.9 | 217.2 | 87.2 | 31.0 | 6.5 |
| NCI-H226 | 345.4 | 99.7 | 374.4 | 99.8 | 19.0 | 12.7 |
| NCI-H2052 | 337.5 | 90.3 | 330.5 | 93.0 | 95.7 | 4.1 |
| NCI-H2452 | 185.0 | 91.3 | 160.9 | 87.3 | 36.4 | 3.2 |

**Suppl. Table 1**: Relative in vitro expression of HER1 in human mesothelioma cells determined by FACS based assay. MFI = mean fluorescence intensity.
